# Effect of breastfeeding education and support intervention (BFESI) versus routine care on timely initiation and exclusive breastfeeding in Southwest Ethiopia: study protocol for a cluster randomized controlled trial

**DOI:** 10.1186/s12887-018-1278-5

**Published:** 2018-09-26

**Authors:** Misra Abdulahi, Atle Fretheim, Jeanette H. Magnus

**Affiliations:** 1 Department of Population and Family Health, Jimma University, Jimma, Ethiopia; 2 Department of Community Medicine and Global Health, University of Oslo, Oslo, Norway; 3 Norwegian Institute of Public Health, Oslo, Norway; 4 Faculty of Medicine, University of Oslo, Oslo, Norway; 5 Department of Global Community Health and Behavioral Sciences, Tulane School of Public Health and Tropical Medicine, New Orleans, USA

**Keywords:** Exclusive breastfeeding, Peer education, Community-based intervention, Peer support, Early initiation of breastfeeding

## Abstract

**Background:**

Infant mortality rates are still high in Ethiopia. Breastfeeding is regarded as the simplest and least expensive strategy for reduction of infant mortality rates. Community-based educational and support interventions provided prenatally and postnatally are effective in increasing breastfeeding rates. However, such interventions are not widely implemented in Ethiopia. This study aims to assess the effect of breastfeeding education and support on timely initiation and duration of exclusive breastfeeding.

**Methods:**

A cluster-randomized controlled trial at the community level will be conducted to compare the effect of breastfeeding education and support versus routine care. The intervention will be provided by Women Development Army leaders who are already in the country’s health system using a 40-h WHO breastfeeding counseling course, “Infant and Young Child Feeding Counseling: an integrated course” and the “Training of Trainers Manual for Counseling on Maternal, Infant and Young Child Nutrition” in the local language. Culturally appropriate operational packages of information will be developed for them. Using preset criteria at least 432 pregnant women in their third trimester will be recruited from 36 zones. Visits in the intervention arm include two prenatal visits and 8 postnatal visits. Supervisory visits will be conducted monthly to each intervention zone. Data will be entered into Epi-data version 3.1 and analyzed using STATA version 13.0. All analysis will be done by intention to treat analysis. We will fit mixed-effects linear regression models for the continuous outcomes and mixed-effects linear probability models for the binary outcomes with study zone as random intercept to estimate study arm difference (intervention vs. routine education) adjusted for baseline value of the outcome and additional relevant covariates. The protocol was developed in collaboration with the Jimma Zone and Mana district Health office. Ethical clearance was obtained from the Institutional Review Board of University of Oslo and Jimma University. This study is partly funded by NORAD’s NORHED programme.

**Discussion:**

We expect that the trial will generate findings that can inform breastfeeding policies and practices in Ethiopia.

**Trial registration:**

ClinicalTrials.gov NCT 03030651 January 25, 2017 version 3 dated 16 July 2018.

**Electronic supplementary material:**

The online version of this article (10.1186/s12887-018-1278-5) contains supplementary material, which is available to authorized users.

## Background

Breastfeeding is a unique way of providing ideal food for the healthy growth and development of infants [1]. Breastfeeding is known to have a beneficial effects in enhancing infants’ immunity, protecting against gastrointestinal and respiratory infections, reducing maternal hemorrhage, as well as the risk of breast and ovarian cancer [2–6]. Breastfeeding is associated with reduced risk of chronic diseases such as diabetes mellitus type 2 [7] and obesity [8–12].

Despite the above benefits to breastfeeding, its prevalence and duration in many countries is below the international recommendation of exclusive breastfeeding (EBF) for the first six months of life. For instance, the proportion of infants less than six months who are exclusively breastfed are 36% globally, 39% in developing countries and 31% in Sub Saharan Africa [13]. A recent systematic review has revealed that risk of all-cause and infection-related mortality was higher in predominantly, partially and non-breastfed infants compared to exclusively breastfed infants aged 0–5 months [14]. Another aspect is timely initiation of breastfeeding within one hour after delivery [15]; early initiation of breastfeeding averages about 43% globally [16]. According to the 2016 Ethiopian Demographic and Health Survey (EDHS) report, only 73% of mothers initiated timely breastfeeding and 58% of children less than 6 months old were exclusively breastfed [17].Moreover, in addition to breast milk, 17% of infants 0–5 months consumed plain water, 5% each consumed non-milk liquids or other milk whereas 11% consumed complementary foods – practices contrary to WHO’s recommendation of EBF. Additionally, 5% of infants under age 6 months are not breastfed at all. The percentage of EBF decrease sharply with age from 74% of infants age 0–1 month to 64% of age 2–3 months and, further, to 36% of infants age 4–5 months [17].

Interventions for breastfeeding promotion have been implemented using different strategies in various settings. At the policy level the extent of conferences, conventions and declarations demonstrate the global efforts in promoting breastfeeding. At the health facility level one of the strategies is the Baby Friendly Hospital Initiative (BFHI). This is a global strategy that promotes breastfeeding in maternity wards around the time of delivery based on the ten steps to successful breastfeeding model [18]; Studies have established the effectiveness of the BFHI-approach in promoting optimal breastfeeding practices particularly in developed countries where the majority of women deliver in health facilities [19–22]. Nevertheless, the effectiveness of the BFHI as well as training of health workers might be limited in developing countries where the majority of deliveries occur at home.

Community- based interventions have been employed in different parts of the world during pregnancy and/or the postnatal period on an individual or group basis, through health facilities or home visiting programmes, using professional education/counselling and peer counselling/support. Most studies on the effectiveness of using peer support/counsellors have reported increased levels of early initiation of breastfeeding and EBF [20, 23–27].

A systematic review of 52 studies from 21 countries revealed that all forms of extra support including lay and professional, analyzed together showed an increase in duration of ‘any breastfeeding’ as well as the duration of EBF. However, the most effective support is provided in person and on a recurring basis at regular scheduled visits [28]. Among breastfeeding promotion interventions involving peer counsellors for support of EBF few studies are from sub-Saharan Africa [29–31].

In Ethiopia, a few behavior change interventions aimed at improving the Infant and Young Child Feeding practice have been conducted by the Non-governmental organizations (NGOs) projects [32–34]. The reports of these projects focus either on implementation fidelity [33], or are implementation research [32] and large scale in scope, focusing not only on breastfeeding but also on other Infant and Young Child Feeding practices [34]. Moreover, none of the interventions were provided during pregnancy as well as the postnatal period and none of the projects used control groups except a trial conducted in Hawassa city [35]. In that trial, the intervention consisted of only one prenatal educational session [35].

The aim of the planned trial is to examine the effect of breastfeeding education and support intervention on timely initiation and duration of EBF in a cluster randomized community based behavioral promotion trial.

### Study objectives/hypotheses

**Research Hypothesis** – Breastfeeding education and support intervention is superior to usual care in improving timely initiation of breastfeeding, exclusive breastfeeding and growth.

**Primary objective -** To determine if breastfeeding education and support intervention is superior to usual care in improving timely initiation of breastfeeding, exclusive breastfeeding and growth.

**Secondary objectives –** the main secondary objectives are

- To validate the Afan Oromo version breastfeeding knowledge and attitude questionnaire.
- To assess baseline knowledge, attitude and practice of mothers on breastfeeding.
- To examine the effect of breastfeeding education and support intervention on mothers’ knowledge and attitude towards breastfeeding.
- To assess the experiences of breastfeeding mothers and WDA leaders participating in the breastfeeding education and support intervention.

## Methods/design

### Design

**Fig. 1:**
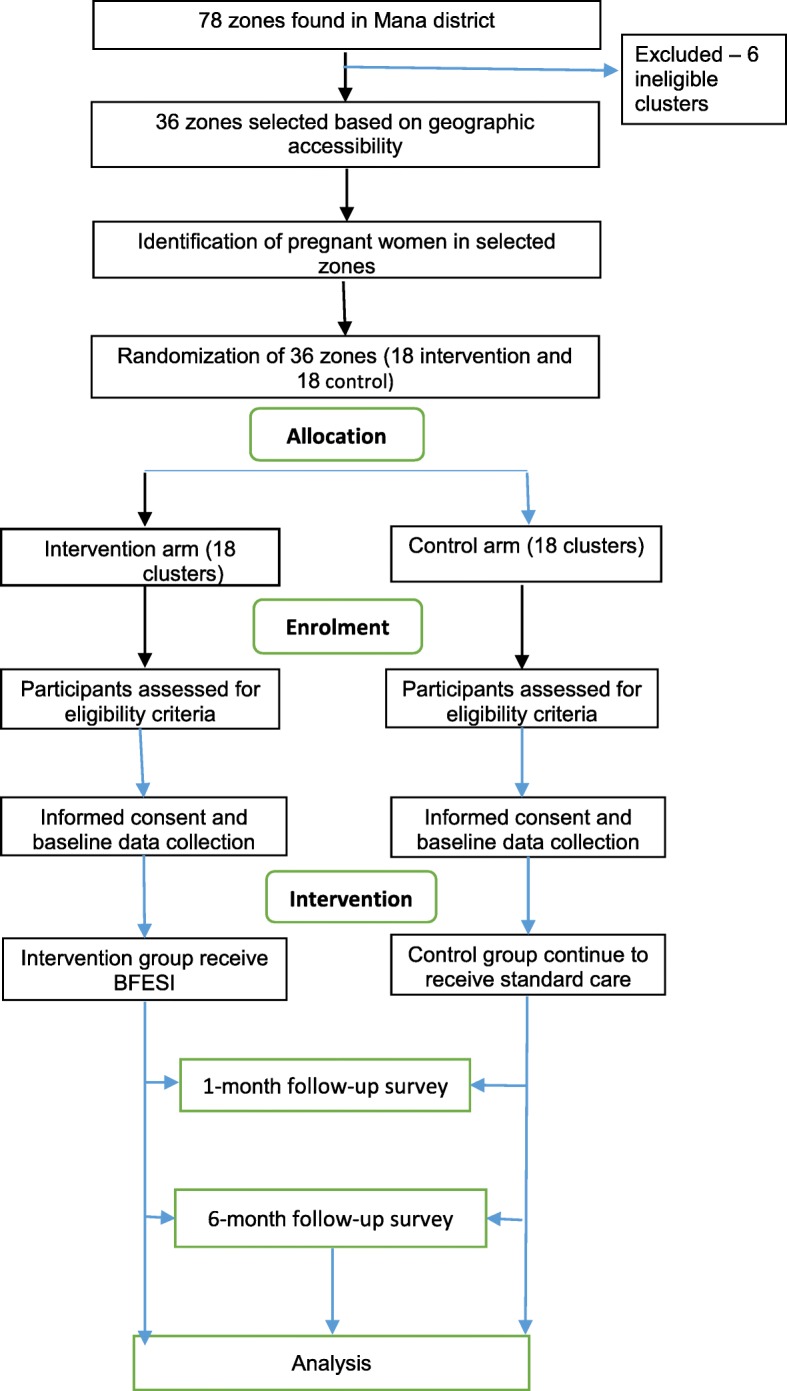
Flow of participants

A cluster randomized controlled single-blind parallel-group, two-arm, superiority trial with 1:1 allocation ratio was designed to investigate whether a breastfeeding education and support intervention provided prenatally and postnatal period increase timely initiation, exclusive breastfeeding duration and infant growth among women in Mana district, Jimma zone, Southwest Ethiopia. This study design was chosen in order to avoid contamination among treatment groups. Clusters are zones found in Mana district, Jimma (Fig. 1).

### Setting

**Fig. 2:**
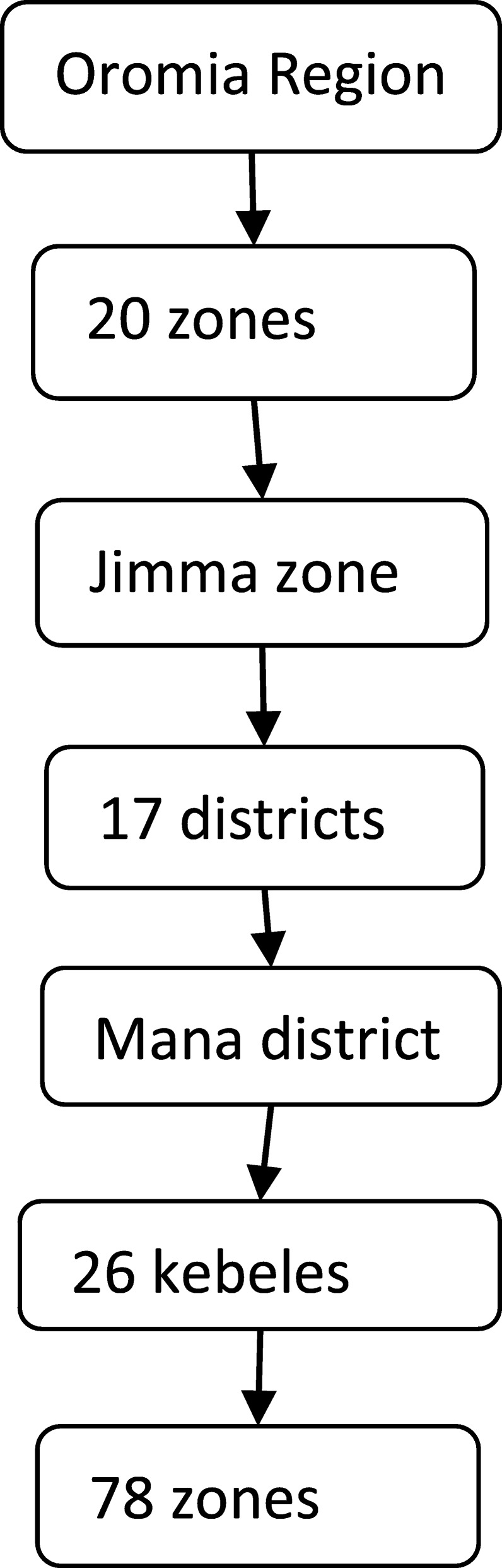
Flow diagram of structures in the Oromia region and the jimma zone

The study will be conducted in the Jimma Zone which is one of 17 administrative zones of the Oromia region, Southwest Ethiopia. Its capital Jimma is situated 352 km to the Southwest of Addis Ababa. Jimma Zone has 17 districts and one special zone. According to population projection of Ethiopia for all Regions at district level from 2014 to 2017, which is based on the 2007 national census, the zone has a total population of 3.1 million in 2016. The rural part accounts for 80.2% of the total population; Oromo is the dominant ethnic group in the area. Health services are provided through 3 hospitals, 112 health centers and 498 health posts. In the Oromia Region a total of 147,428 Health Development Army (HDA) groups and 732,259 one-to-five networks were established in 2011 [36]. The one-to-five networks are women volunteers who are empowered as a HDA to transform their society. They are trained to focus more intensively on sparking local behavior change making regular rounds to check on neighbors and encourage practices like latrine building and setting-up separate cooking spaces. They are from “model families” and serve as living examples that the health extension workers’ messages are being heard [37]. The proportion of women of child bearing age is 24% [38]. The trial will be conducted in the Mana district which is one of the 17 districts found in the Jimma zone. The district has 26 kebeles - the lowest administrative unit and each kebele is divided into three small zones (Fig. 2).

### The context

#### Health extension program

As part of an accelerated primary health care expansion to the community the government of Ethiopia launched its health extension program (HEP), an innovative community-based strategy to deliver preventive and promotive services, and selected high impact curative interventions at community level in 2003. In addition to the construction of health posts and provision of supplies, the implementation strategy of HEP focused on building human resource by deploying two salaried female Health Extension Workers (HEWs) at health posts in each kebele (village) of the country [39]. All HEW trainees are women aged 18 and above with a minimum of 10th grade schooling. In order to increase acceptance, these HEWs are drawn from the communities in which they serve. They complete a one year training of courses and field work that is provided by Technical and Vocational Education Training Schools (TVETs), operated by the Ministry of Education. Upon completion, two HEWs are assigned to each health post which serves as the basis for the HEP [40].

HEWs promote the 16 health packages in the HEP consisting of disease prevention and control, family health, hygiene and environmental sanitation as well as health education and communication [40]. HEWs implement the health promotion program through house to house visits. The interventions include: promotion and provision of contraceptives, antenatal care including nutritional advice and micronutrient supplementation, clean delivery, basic new-born care, child nutrition (such as exclusive breastfeeding, complementary feeding, cooking nutritious meals, and vitamin A supplementation), immunization, use of mosquito bed nets, HIV prevention, sanitation, and hygiene (including support and supervision in the construction of latrines, disposal pits and healthful homes) [41]. HEWs spend 75% of their time visiting families in their homes implementing promotional and preventive interventions to create appropriate healthy behaviors and to improve knowledge and attitude toward health-seeking behaviors.

The remaining 25% of their time is spent providing the following services at the health post: immunization, health education, antenatal care, family planning; delivery and postnatal care, growth monitoring of children, community treatment of severe acute malnutrition, diagnosis and treatment of malaria, diagnosis and treatment of pneumonia, treatment of diarrhoea with oral rehydration fluids, treatment of eye infections with eye ointment, treatment of selected skin problems with ointments, Vitamin A supplementation, first aid, referral of difficult cases, documentation, and reporting. The HEWs’ community outreach activities include promoting model families, community groups or households [41, 42]. At least two diploma level midwives and one health officer with emergency obstetric care training support HEWs from the local health center [43].

The HEW selects “model families” in collaboration with the village administration. Model families are households who receive 96 h of training and adopt all 16 HEP packages [41] – from vaccinating their children and sleeping under mosquito bed-nets to building separate latrines and using family planning [42]. The training involves face-to-face teaching and household visits in four modules corresponding to the four HEP subprograms: prevention of communicable diseases, family health, environmental and household sanitation, and health education. Model families are expected to disseminate their knowledge and behavior to other households in order to support the HEWs’ efforts [44].

#### Antenatal and postnatal care

Within their catchment area, HEWs are responsible for identifying pregnant women, providing antenatal care (ANC) and connecting them with the formal health system in the event of elevated risk or complications. They provide four focused ANC visits throughout a woman’s pregnancy using an integrated maternal and child care card. Women see the same HEW for all four home visits: first visit after 16 weeks of pregnancy, second visit between weeks 24–28, third visit between weeks 30–32 and fourth visit between weeks 36–40. The HEW conducts a general physical examination and evaluation at each visit, checking the mother and the growth of the foetus. HEWs also assess all pregnancies for the potential risks by communicating with women and their families about the danger signs of complications so that there is a shared responsibility for identification and action when needed. Furthermore, HEWs develop an individualized birth preparedness and complication readiness plan with each woman, involving the women’s partner or support whenever possible [43].

During delivery, the same HEW is able to assist by accompanying a woman to a health facility for delivery. HEWs are trained in pre-referral clinical procedures such as starting intravenous fluids and catheterization [45]. After delivery, HEWs do follow up visits during the postnatal period when care is critical for both mother and new-born. The initial postnatal care visit occurs ideally within four hours of delivery [46]. They conduct the next follow-up postnatal visits at two days, six days, and six weeks [43].

#### Women development Army (WDA) groups

In 2011 the government started the Health Development Army (HDA) with the aim to consolidate the gains made as a result of roll out of the HEP and promote community ownership of the programs. The program was first tested in Tigray and then introduced to the four big regions of the country. Although some regions have both male and female HDAs, HDAs are now basically women known as the women development army (WDA) [38].

WDAs are identified from the model families. As soon as the WDA groups are formed through a participatory community engagement, the WDA leaders go through an intensive 7 to 10 days training program [38], whose primary task is to educate and mobilize communities to use available high impact maternal, neonatal and child health (MNCH) services provided by the health post and health centres [42]. In a kebele of 1000 households, averages of 150 leaders go through the training program that is supported by the local PHC unit and the woreda (district) health office. In an average kebele, there are approximately 30 WDA team leaders and 200 WDA network leaders [38].

**Fig. 3:**
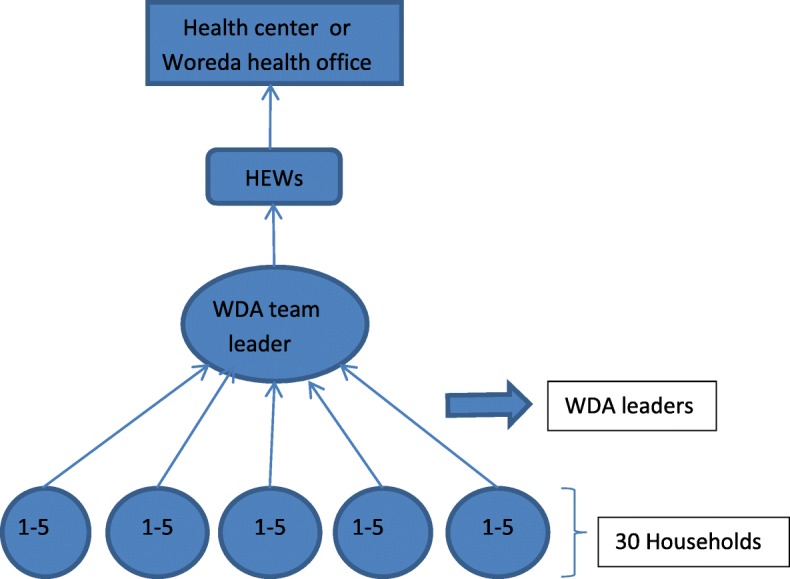
Hierarchy of reporting

Each WDA group consists of 25–30 households (women) which are further organized into the “1 to 5” network of women where a model woman leads five other women within her neighbourhood [47]. Designed to empower women in particular and the family in general in health decision making leading to democratization of health and to community partnership, the one-to-five network functions as a forum for exchange of concerns, priorities, problems and decisions related to the health status of women. While being supported by the HEWs the networks are responsible for the preparation of plans and ensuring their completion, for the collection of health information, and also for conducting weekly meeting to review progress and submitting monthly reports [41]. The WDA groups thus support the implementation of the HEP (Fig. 3).

The one-to-five networks meet every week, while the larger health development team meets once every two weeks. Furthermore, they review their performance against their plan and evaluate each other on monthly basis and give grades A, B, and C for top, middle and poor performers, respectively. A performance report including the grades is organized at the health development team level and sent to the HEWs [38].

#### Eligibility criteria for clusters and WDA leaders

Out of 78 zones found in Mana district, 36 clusters that are not adjacent to each other and have geographical accessibility will be selected randomly for the study - 18 intervention and 18 controls.

Women development army leaders, one from each selected zone, who are influential members of their community will be selected by maternal health focal person at Mana district health office.

#### Eligibility criteria for participants

Participants for this trial will be healthy pregnant women in their third trimester. We will recruit them using a 2-stage screening process. An initial screening will take place while women are pregnant; the second screening will take place after delivery to ascertain whether both mother and infant are qualified for inclusion. Inclusion criteria during pregnancy will be pregnant women in the third trimester, living in the selected cluster with no plans to move away during the intervention period, without psychiatric illness, capable of giving informed consent and willing to be visited by supervisors and data collectors. Inclusion criteria after delivery will be a singleton live birth with no severe malformation that could interfere with breastfeeding. Exclusion criteria will be maternal death, women with severe psychological illness which could interfere with consent and study participation, severely ill or clinical complications warranting hospitalization, stillbirth, infant death, twin gestation, or preterm birth (at < 37 weeks gestation).

#### Sample size determination

Sample size was calculated using Sample Size Calculator (SSC) a Windows based software package [48] with the following assumptions: to detect an increase in exclusive breastfeeding for 6 months from 58 to 78% [17], with 95% CIs and 80% power, assuming an intra-cluster correlation coefficient of 0·1 equal to the Ugandan study [49] for a cluster size of 10, it was calculated that we will need 36 clusters. Adding 20% of the sample size for loss to follow-up, the final sample size is 432 pregnant women (216 in intervention, and 216 in control groups).

#### Sampling and randomization procedures

Zones in kebeles will form the unit of randomization for the trial, while mothers within the zones will form units of observation. From the 17 districts in Jimma zone, one district will be selected purposively after excluding districts with similar ongoing intervention or project. After identifying and listing the 78 zones found in the selected woreda, 36 non adjacent zones will be selected. Then eligible pregnant women will be identified from the selected zones using Health Extension Worker’s logbook before the zones are randomized into either treatment group. A simple randomization with a 1:1 allocation will be used to randomize zones to either control or intervention group. First, the 36 zones will be listed alphabetically and then a list of random numbers will be generated in MS Excel 2010 and the generated values will be fixed by copying them as “values” next to the alphabetic list of the zones. These will then be arranged in ascending order according to the generated random number. Finally, the first 18 zones will be selected as intervention clusters and the last 18 as control clusters.

A statistician that is blinded to study groups and not participating in the research will do the generation of the allocation sequence and the randomization of clusters. Allocation Concealment will not be done for study participants, as they will certainly know if they were in the intervention group or not. Data collectors will be masked to the zone allocation by not informing them of the allocation, not making them part of trial implementers and not being residents in any of the zones.

#### Recruitment

Before cluster randomization, all pregnant women in the randomized zones will be identified by reviewing Health Extension Worker’s logbook. However, recruitment will be started after clusters have been randomized. During recruitment, WDA leaders will additionally be used to identify pregnant women in their 1–5 network to minimize the chance of missing any pregnant women in each cluster zone. Identified pregnant women will be invited to a meeting at the health post where the nature and purpose of the trial and eligibility criteria will be explained.

Informed consent will be obtained from each woman prior to their inclusion in the trial. Verbal consent will be obtained to ensure approval by the woman that she could be visited by WDA leaders for the intervention. Then the written consent will be obtained from all women who will be enrolled into the study. The data collectors will explain all trial procedures from inclusion criteria to the last follow-up using an information sheet. The women will be allowed to ask questions and relevant information will be provided accordingly. The data collectors will be trained using simulation situations. Women who are willing to consent will either sign or put their finger-prints according to their literacy status. If a woman declines, a form will be filled and she will be thanked. All women in the intervention clusters will receive the breastfeeding education and support if they wish to do so, whether they participate in data collection or not. To retain study participants with complete follow up there will be repeated visits. Unless a clear reason for non-participation at a scheduled visit is given, three attempts to visit the mother-infant pair will be made before a visit is considered as missed. A recruited mother will be revisited until the last scheduled visit, irrespective of the number of missed visits, unless there is a clear reason for termination. Regardless of decision to discontinue their assigned intervention, study participants will be retained in the trial whenever possible to enable follow up data collection and prevent missing data. All pregnant women in the randomized clusters will be identified and approached in order to minimize selection bias. Participants are enrolled from May to August 2017.

The assigned study intervention may need to be discontinued for a given trial participant if there is withdrawal of participant consent. As part of the need for intervention modification, additional visits will be arranged for both educational and practical support intervention for study participants whenever there is missed visit. Strategies to improve and monitor adherence include repeated breastfeeding education and support intervention as well as repeated outcome measurement whenever there is missed visit. There can be a possibility of exclusive breastfeeding information through mass media that participants may not be prohibited to follow.

#### Training of supervisors and women development Army leaders

Although WDA leaders are acknowledged from a model family, they need to extend their knowledge and skills through appropriate training and support to become effective peer counsellors/support. Therefore, the 18 WDA leaders from the selected intervention clusters will be trained as peer supporters together with the supervisors for five days at Mana District Health Office using the WHO/UNICEF “Breastfeeding Counselling Course”, “Infant and Young Child Feeding Counseling: an integrated course” and the “Training of Trainers Manual for Counseling on Maternal, Infant and Young Child Nutrition” [50–52]. A trainer’s and participants’ manuals will be developed based on the above three training materials. Both the trainer’ and participants’ manuals will be translated to local language (Afan Oromo) by language expert and a health professional who is nutritionist will review the translation. Accordingly adjustment will be made to the manual considering the local culture. As some WDA leaders may have writing and reading skills in Amharic (national language), this will be identified ahead of time and a participant’s manual will be prepared in Amharic. During the training, methods proposed in the above manuals will be used. The training has three parts: classroom sessions for providing theoretical aspects of breastfeeding, counselling and communication; practical sessions on counselling skills (listening and learning skills, confidence and support skills) and supervised fieldwork with pregnant and lactating mothers. The following teaching methods will be used during the training: lectures, demonstrations, clinical practice, and work in smaller groups with discussion and role-plays.

#### Classroom sessions

Classroom sessions will include lectures and interactive discussions on the benefits of breastfeeding, benefits of timely initiation of breastfeeding, disadvantages of prelacteal feeds and bottle-feeding, benefits of exclusive and frequent breastfeeding, how breastfeeding works (anatomy and physiology of breast), positioning and latching on, assessing a breastfeed, counselling (listening, learning, building confidence and giving support), identification and management of breast problems, refusal of breastfeed, taking a breastfeeding history, breast examination, expressing breast milk, identification and management of breastfeeding problems, importance of the mother’s diet during pregnancy and lactation and use of lactational amenorrhea method (LAM) and other family planning options. During the classroom sessions, the cultural norms of the community will be explored not to violate their cultural practice. Once the cultural norms are identified, the training will be given keeping the essence of the intervention by respecting their norm. Moreover, for those trainees who choose a working local language other than Afan Oromo, their concern will be taken into account. Trainers will also make sure that WDA leaders understand the lectures using both Amharic and Afan Oromo language if need arise.

#### Practical sessions

WDA leaders will be taught about different skills through demonstrations and role plays. The skills will include: listening to mothers and learning about their problems, assessing position and latching of babies during a breastfeed, building mothers’ confidence and giving support, identification and management of breast problems, taking a breastfeeding history, breast examination, expressing breast milk, identification and management of breastfeeding problems and providing relevant information and practical help when required. During the demonstrations and role plays, the trainers will make sure that WDA leaders respect the cultural norms of the community and use a language the women understand.

#### Field work

Ten pregnant mothers and another ten women with recent deliveries will be “enrolled” for the practice and counselling. During this visit, 4 to 5 WDA leaders will form one group to avoid overcrowding a room with a new-born. Counselling will be provided by one of the WDA leaders whereas others will observe and complement as needed. The supervisor will interfere only if the counselling is incomplete. During the field work, trainers will ensure that WDA leaders respect the cultural norms of the community and use a language the women understand.

#### The intervention description

##### Control group

For this trial a standard/usual care is chosen as a comparator for the breastfeeding education and support intervention as per the World Medical Association (WMA) of Helsinki declaration. Women in the control group will receive the routine health and nutrition education during prenatal and postnatal period that is currently offered to mothers by HEWs and WDA leaders working in their cluster.

The standard/routine prenatal and postnatal care by HEWs and WDA leaders in Ethiopia include: WDA leaders educate and mobilize communities to use available high impact maternal, neonatal and child health (MNCH) services provided by the health post and health centres, whereas HEWs provide four focused ANC visits, develop an individualized birth preparedness and complication readiness plan with each woman, accompany a woman to a health facility during delivery and conduct 4 postnatal visits.

##### Intervention group

Women in the intervention group will receive an enhanced breastfeeding education and support intervention from third trimester during pregnancy till 5 months post-delivery. The intervention is composed of the following elements: a) prenatal breastfeeding education to raise knowledge and awareness where benefits of breastfeeding will be emphasized, b) postnatal breastfeeding counselling and support.

After being trained, WDA leaders will provide breastfeeding education and support through a house to house visit before and after delivery. Besides the routine information and education HEWs and WDA leaders provide to the women, each visit will be designated to cover specific topics related to the outcomes of the study.

##### Education and support by peer-supporters during pregnancy

WDA leaders will function as peers for mothers in their clusters. During each visit WDA leaders will, in addition to a specific topic from the health package, cover in detail the importance of timely initiation of breastfeeding and EBF, feeding colostrum first, and discouraging prelacteal and postlacteal foods and encourage the mother to deliver at the nearby health center. The discussion will be combined with use of educational materials and practical demonstrations on proper breastfeeding positioning and attachment. Mothers will be encouraged to ask any question related to topics discussed.

WDA leaders will use language and culturally appropriate visual educational materials in the form of flip charts to illustrate the new information (e.g., correct and incorrect breastfeeding positions, correct and incorrect breastfeeding latching on, examples of how the father/significant others can support the mother with breastfeeding), and the benefits of applying this new information to practice (e.g., pictures of babies who were breastfed versus those who were not).

##### Visits after delivery

During the first two weeks after delivery, WDA leaders will visit the mothers in their group on days 1 or 2, 6 or 7 and 15th day and encourage them to breastfeed frequently and on demand and to stop prelacteals and postlacteals if these have been given. During each visit, mothers will be observed positioning, latching on, and feeding the new-born, with appropriate feedback provided, solving any BF problems, emphasize nutrition for sufficient breast milk to breastfeed successfully and hands-on guidance only when necessary. They will support and encourage the mothers to continue exclusive breastfeeding for 6 months. WDA leaders will also promote personal cleanliness and domestic hygiene, and hand washing before feeding, after going to the toilet, and after changing babies’ diapers.

##### Monthly visits

Starting from the 1st month of delivery, the mothers will be visited monthly for the first five months postpartum. During these visits mothers will be observed positioning, latching on, and feeding the newborn, with appropriate feedback provided, emphasizing techniques for preparing for work and management of breast milk (breast milk expression, storing breast milk), encouraging the mothers to continue exclusive breastfeeding for 6 months, discuss lactational amenorrhea method (LAM) and other family planning options, providing hands-on guidance only when necessary. WDA leaders will also stress personal cleanliness and domestic hygiene, and hand washing before feeding, after going to the toilet, and after changing babies’ diapers.

##### Additional visits and referral

If a baby or mother becomes sick, family members will inform the WDA leader and the WDA leader will inform the situation to the HEWs. Then the HEW will make a visit to that household to identify the problem and provide the necessary care. If there is an urgency or if the situation do not improve within 2 days, she will make referral to the next level.

##### Supervisors

Two persons who are currently involved in the supervision of the HEWs and participated in the WHO training with the WDA leaders will serve as supervisors. The breastfeeding supervisors’ main responsibility will be to provide supportive supervision and monitor the WDA leaders. Supervisory visits will be conducted by the researcher along with supervisors monthly. WDA leaders will receive feedback on their work from the supervisors during monthly supervision meetings.

##### Outcome assessment

**Primary outcomes** of the trial include timely initiation of breastfeeding, exclusive breastfeeding at 6 month and infant growth**.**

**Timely breastfeeding initiation** is measured as the proportion of women who initiated breastfeeding her baby within the first hour after delivery.

**Exclusive breastfeeding at 6 month** is measured as the proportion of women who provided their infants with only breast milk but no solids, nonhuman milk, water, or other liquids (other than vitamins or medications) at six months.

**Infant growth** - WHO Child Growth Standards (2006) will be used to estimate anthropometric status at 6 month [53]: weight-for length z-scores (WLZ), length-for-age z-scores (LAZ) and weight- for-age z-scores (WAZ). Children who have WLZ below− 2 (WLZ < − 2) will be considered wasted, those with LAZ below− 2 (LAZ < − 2) stunted, and those with WAZ below − 2 (WAZ < − 2) underweight.

**Secondary outcomes are** validation of the Afan Oromo version breastfeeding knowledge and attitude questionnaire, baseline knowledge, attitude and practice of mothers on breastfeeding, change in mothers’ knowledge and attitude towards breastfeeding at baseline and study completion, mothers' and WDA leaders' experiences of the intervention at study completion.

Outcomes will be assessed as illustrated in the Standard Protocol Items: Recommendations for interventional trials (SPIRIT) (Table 1).

**Table 1:** Standard Protocol Items: Recommendations for Interventional Trials (SPIRIT)

Outcomes	Allocation	Study period			
		Enrollment		Close-out	
	-t_1_	Baseline (t_0_)	Intervention (8 months)	Midline at month 1 after delivery (t_1_)	End line at month 6 (t_2_)
Enrollment					
Allocation	x				
Eligibility screen		x			
Informed consent		x			
Interventions					
BFES Intervention			x		
Control group			x		
Assessments					
Background/demographics		x			
Water, Hygiene and Sanitation		x			
Household Food Insecurity Access Scale		x			
Food Taboos during Pregnancy & Lactation		x			
Mother’s Health Status & Obstetric history		x			
Current pregnancy and breastfeeding		x			
Timely breastfeeding initiation				x	
Exclusive breastfeeding at 6 month					x
Infant growth					x
Validation of the Afan Oromo version breastfeeding knowledge and attitude questionnaire			x		
Baseline knowledge, attitude and practice of mothers on breastfeeding			x		
Change in mothers’ knowledge and attitude towards breastfeeding at baseline and study completion			x		x
Mothers' and WDA leaders' experiences of the intervention at study completion					x

##### Data collection tools and techniques

Ten data collectors will be recruited and trained for 2 days. A structured questionnaire prepared in Afan Oromo will be used to collect data. Components in the questionnaire will be prepared by adapting tools validated for use in similar contexts. Data will be collected at baseline, 1st month and 6th month. Data collection interviews will be made 1–3 days before counselling visits. Data on socioeconomic and demographic variables, maternal and pregnancy factors, and previous infant feeding experience will be collected at baseline. Information on delivery, about early initiation, whether colostrum was discarded, use of prelacteals, and reasons for delaying or not initiating breastfeeding will be obtained one month after birth. Data on knowledge and attitude will be collected at baseline and at study completion. Anthropometric measurements (length, weight and mid upper arm circumference (MUAC)) will be done at 6 month. Length will be measured using length board at a precision of 0.1 cm. Infant’s weight will be measured to the nearest 1.0 g using UNICEF SECA weighing scales with light clothing. MUAC will be measured to the nearest 0.1 cm on the left arm using non- stretchable MUAC tape. Length and MUAC measurements will be done in duplicate. The measurement procedures will follow standard WHO guidelines [54]. All data collectors will be trained on content, questionnaire techniques and measurements and will be kept uninformed about cluster allocation. Reproducibility and validity exercises will be conducted for the weight and length measurements. Mothers’ and WDA leaders’ experience about the intervention will be assessed qualitatively at study completion. To obtain feedback on the intervention, individual semi-structured interviews will be held with intervention mothers at the end of 6 months after end-line data is collected. Additionally, focus group discussions will be carried out with WDA leaders to assess their opinions about the intervention strategy. All group discussions will be moderated by the principal investigator. Another person will take notes while the discussions are simultaneously audio-taped. Additionally, a field memo will be completed for each FGD and interview, and socio- demographic data will be collected for all participants based on a brief questionnaire (e.g., age, education, etc.). The memos will include observations about the group dynamics, information about non-verbal responses, questions that elicited hesitations or any interruptions or difficulties in running the focus groups. Breastfeeding Knowledge and Attitude (B-KA) questionnaire will be developed and adapted using the following validated instruments: the Iowa Infant Feeding Attitude Scale (IIFAS) [55] and the Breastfeeding Knowledge Questionnaire (BKQ) [56].

##### The Iowa infant feeding attitudes scale (IIFAS)

The Iowa Infant Feeding Attitudes Scale (IIFAS) was developed in 1999 by de la Mora [55]. The scale was designed to assess maternal attitude toward infant feeding methods and to predict breastfeeding intention and exclusivity. It is composed of 17 items with a 5-point Likert scale ranging from 1 (strongly disagree) to 5 (strongly agree). Eight items are worded in manner favoring breastfeeding, whereas the other 9 favors bottle feeding. Items favoring bottle-feeding are reverse scored. The total IIFAS score ranges from 17 (reflecting positive attitudes toward bottle-feeding) to 85 (reflecting more positive attitudes toward breastfeeding). It has been validated in different countries such as the United States [55], Northern Ireland [57], Scotland [58], Japan [59], Romania [60] and China [61].

##### Breastfeeding knowledge questionnaire

The questionnaire assessing breastfeeding knowledge was adapted and modified from a breastfeeding questionnaire developed by a team of pediatric nurses at the Hospital Universiti Sains Malaysia (HUSM) [58]. Items in the modified questionnaire covered the following scopes of knowledge on breastfeeding: general knowledge, colostrum, advantages to mothers and babies, effective feeding method, duration of feeding, expressed breast milk (EBM), storage of EBM, complementary feeding, and problems with breastfeeding.

##### Instrument translation

Permission to use both instruments will be obtained from authors. A systematic process recommended by Beaton et al. [62] will be used for maintaining semantic, idiomatic, experiential and conceptual equivalence. The instruments will be forward translated to Afan Oromo by bilingual translators. Two bilingual translators who are totally blind to the original English version will translate the instruments back to English. The back-translation is essential to establish semantic equivalence. An expert committee meeting will be held to consolidate all versions of translation to develop the pre-final version of the questionnaire for field testing.

The original and the back-translated English version of instruments will be compared to check for the accuracy of translation. A method developed by Skperber et al. [63] will be used for validating the translated instrument where each item in the original and back-translated versions will be ranked in terms of comparability of language and similarity of interpretability. Ranking will be done by raters who are fluent in English using Likert scales ranging from1 (extremely comparable/extremely similar) to 7 (not at all comparable/not at all similar). Any mean score > 3 requires a formal review of the translation. The translated instruments will then be piloted among 30 pregnant women to assess its clarity, comprehension, length, and cultural acceptability. The questionnaires will finally be used to assess knowledge and attitude of study participants who will be enrolled into the trial at baseline and completion of the study.

##### Data management

All filled questionnaire will be checked for completeness by supervisors and questionnaires with missing items will be returned to data collectors for correction. Participants who are lost to follow up will be recorded along with their reasons. The following standard processes will be implemented to improve the accuracy of data entry and coding: double data entry; verification that the data are in the proper format (eg, integer) or within an expected range of values; and independent source document verification of a random subset of data to identify missing or apparently erroneous values. Only the study team will have access to the trial datasets. In order to ensure confidentiality, information about each zone and personal data of participants will not be shared with any third party both during and after the trial. Additionally, all personal identifiers will be removed from database and filled questionnaires will be stored in a locked cabinet. Data will be stored in a database on a password protected computer and only accessible by the study team.

##### Data processing and analysis

Double data entry will be performed using EpiData version 3.1 (EpiData Association) and consistency checks and statistical analysis will be done using Stata version 13.1 (StataCorp). The effect of the intervention in comparison with the routine education will be assessed by fitting mixed-effects linear regression models for the continuous outcomes and mixed-effects linear probability models for the binary outcomes using study zone as random intercept to account for clustering of subjects by zones. The use of linear probability model for binary outcomes is well-established and allows for a straightforward interpretation of the average intervention effect expressed as risk difference using percentage points [64]. Fixed-effects in the models will include study arm (intervention vs. routine education), baseline value of the outcome when appropriate, and additional covariates that could potentially confound estimation of group difference. Potential covariates will be selected based on consideration of study arm balance at baseline and potential confounders for an outcome previously reported in the literature. Potential confounders of timely breastfeeding initiation include: place of delivery, mode of delivery [65] whereas potential confounders of EBF are mode of delivery, age, parity, educational status, previous BF experience, household income and breastfeeding intention [30, 65–68]. We will further explore effect modification of the intervention by the different covariates by adding interaction terms between study group and a covariate as fixed-effect parameter. Subgroup analysis of the intervention effect by a covariate will be considered when there is significant interaction (*P* < 0.1) between the intervention and a covariate. Moreover, mean values of anthropometric data of infants in the intervention and control groups will be compared using Student’s t test and linear regression will be used. In all analyses, adjustment will be made for clustering at the zone level since randomization was done at cluster level rather than individual level.

All analyses will be performed by the intention-to-treat principle including all subjects initially enrolled into the study. For this purpose, we will conduct a multiple imputations procedure for the missing data using chained equations under the missing at random assumption. Fifty imputations of missing data will be generated to estimate the regression coefficients. All tests will be two-sided and the level of significance will be set at alpha < 0.05. No interim analyses are planned and all outcomes will be analyzed after data collection is completed.

All FGDs and interview audio files and notes will be translated to English, then transcribed verbatim and analyzed thematically using Systematic Text Condensation, a descriptive and explorative analysis strategy in Atlas Ti7 software. Malterud’s text condensation follows four steps: 1) total impression - from chaos to themes; 2) identifying and sorting meaning units - from themes to codes; 3) condensation - from code to meaning; 4) synthesizing - from condensation to descriptions and concepts. It is a qualitative research method particularly suited to analyze qualitative data, such as interview studies, observational studies and the analysis of written texts [69].

##### Data monitoring

Since the risks of harm are small, the study does not have a data monitoring committee. Moreover, earlier stopping points cannot be anticipated as effectiveness will not be able to be determined until the end of the study.

##### Dissemination

To make the trial transparent, the full protocol will be published on open access journal. However, participant- level dataset, and statistical code will not be accessed publicly for the sake of ensuring confidentiality. Any modifications that will be made to the protocol will be communicated to relevant parties such as trial registry and the ethics committee. Articles from this research project will be published in peer reviewed journals. Result of the study will be presented to Jimma zone health office. Moreover, findings will be presented at national and international conferences and workshops. Authorship eligibility guidelines and any intended use of professional Writers. ICMJE guideline for authorship will be followed.

The protocol adheres to the recommendations provided by the SPIRIT 2013 (Additional file 1). Moreover, all items from the WHO Trial Registration Data Set are available (Additional file 2).

## Discussion

At the beginning, the aim was to include all pregnant women in selected clusters who are in their third trimester. However, when recruitment was started, we failed to get intended number of women in each cluster. As one of the purposes of using cluster randomized controlled trial is to prevent information contamination among study participants, we could not increase number of clusters beyond 36 and also there was a logistic constraint to expand the trial into another district. Therefore, we included women who are in their second trimester to fill our sample size; however, the intervention for these women will start when they are in their third trimester. This will inevitably prolong the intervention duration for a few months. If this intervention proves to be effective in improving the timely initiation and exclusive breastfeeding rates, it will be scaled up in other parts of Oromia region and other regions of the country.

### Trial status

From the 17 districts found in Jimma zone, Mana district is selected purposely. The 36 zones selected from 78 zones found in Mana district are randomized. Trainers’ and participants’ manual was prepared in local language (Afan Oromo and Amharic). Training of intervention implementers (Women Development Army leaders) in the intervention arm is completed and visual teaching material, flip chart, is prepared in both Afan Oromo and Amharic language. All trial participants have been recruited form the selected clusters and baseline data are collected. Data at month one (for timely initiation of breastfeeding) has already been collected while data collection for exclusive breastfeeding and other outcomes at month 6 has started.

## Additional files

Additional file 1: SPIRIT 2013 Checklist for the BFESI cRCT. (DOC 124 kb)

Additional file 2: Items from the WHO Trial Registration Data Set. (DOCX 14 kb)

## Abbreviations

BF: Breastfeeding
BFHI: Baby Friendly Hospital Initiative
CI: Confidence Interval
EBF: Exclusive Breastfeeding
EDHS: Ethiopian Demographic and Health Survey
FGD: Focus Group Discussion
HDA: Health Development Army
HEWs: Health Extension Workers
UNICEF: United Nation Children’s Fund
WDAs: Women Development Army
WHO: World Health Organization
