# Supplementary material for: Effect of breastfeeding education and support intervention (BFESI) versus routine care on timely initiation and exclusive breastfeeding in Southwest Ethiopia: study protocol for a cluster randomized controlled trial
Source: BMC Pediatr. 2018 Sep 26;18:313. doi: 10.1186/s12887-018-1278-5 (PMC6158863; doi:10.1186/s12887-018-1278-5)
Supplement: Supplementary file 2 — Items from the WHO Trial Registration Data Set. (DOCX 14 kb) [file 12887_2018_1278_MOESM2_ESM.docx]

| **Category** | **Information** |  |  |
| --- | --- | --- | --- |
| **Primary Registry and Trial Identifying Number** | ClinicalTrials.gov NCT03030651 |  |  |
| **Date of Registration in Primary Registry** | January 2017 |  |  |
| **Secondary Identifying Numbers** | None |  |  |
| **Source(s) of Monetary or Material Support** | NORAD under NORHED programme |  |  |
| **Primary Sponsor** | University of Oslo |  |  |
| **Secondary Sponsor(s)** | Jimma University |  |  |
| **Contact for Public Queries** | MA, BSc, MPH [misra_ab@yahoo.com] |  |  |
| **Contact for Scientific Queries** | MA, BSc, MPH, University of Oslo, Norway |  |  |
| **Public Title** | Breastfeeding education and support intervention to optimize breastfeeding practice for improving infant growth |  |  |
| **Scientific Title** | Effect of Breastfeeding Education and Support Intervention versus routine care on Timely Initiation and Exclusive Breastfeeding in Southwest Ethiopia: study protocol for a cluster randomized controlled trial, BFESI |  |  |
| **Countries of Recruitment** | Ethiopia |  |  |
| **Health Condition(s) or Problem(s) Studied** | Breastfeeding (Exclusive), Nutrition Status |  |  |
| **Intervention(s)** | Breastfeeding Education and Support Intervention |  |  |
| **Key Inclusion and Exclusion Criteria** | Inclusion criteria during pregnancy will be pregnant women in the third trimester, living in the selected cluster with no plans to move away during the intervention period, without psychiatric illness, capable of giving informed consent and willing to be visited by supervisors and data collectors. Inclusion criteria after delivery will be a singleton live birth with no severe malformation that could interfere with breastfeeding. Exclusion criteria will be maternal death, women with severe psychological illness which could interfere with consent and study participation, severely ill or have clinical complications warranting hospitalization, stillbirth, infant death, twin gestation, or preterm birth (at <37 weeks gestation). |  |  |
| **Study Type** | Interventional |  |  |
|  | Method of allocation – random, Masking is used - data collectors are blinded, Assignment – parallel |  |  |
|  | Purpose – Behavioural promotion |  |  |
| **Date of First Enrolment** | May 2017 |  |  |
| **Target Sample Size** | 432 |  |  |
| **Recruitment Status** | Recruitment completed |  |  |
| **Primary Outcome(s)** | Timely initiation of breastfeeding, Exclusive Breastfeeding and Growth |  |  |
| **Key Secondary Outcomes** | Validation of the Afan Oromo version breastfeeding knowledge and attitude questionnaire, baseline breastfeeding knowledge and attitude, Change in knowledge and attitude at baseline and endline, Women’s and WDA leaders’ experience at endline |  |  |
|  |  |  |  |
|  | | |  |
|  | | |  |
|  | | |  |
|  | | |  |
|  | | |  |
|  | | |  |
|  | | |  |
